# Liver cirrhosis, other liver diseases, and risk of hospitalisation for intracerebral haemorrhage: A Danish population-based case-control study

**DOI:** 10.1186/1471-230X-8-16

**Published:** 2008-05-24

**Authors:** Henning Grønbæk, Søren P Johnsen, Peter Jepsen, Mette Gislum, Hendrik Vilstrup, Ulrik Tage-Jensen, Henrik T Sørensen

**Affiliations:** 1 Department of Medicine V, Aarhus University Hospital, Denmark; 2 Department of Clinical Epidemiology, Aarhus University Hospital, Denmark; 3 Dept. Medical Gastroenterology (Medical Center), Aalborg, Denmark

## Abstract

**Background:**

Liver diseases are suspected risk factors for intracerebral haemorrhage (ICH). We conducted a population-based case-control study to examine risk of ICH among hospitalised patients with liver cirrhosis and other liver diseases.

**Methods:**

We used data from the hospital discharge registries (1991–2003) and the Civil Registration System in Denmark, to identify 3,522 cases of first-time hospitalisation for ICH and 35,173 sex- and age-matched population controls. Among cases and controls we identified patients with a discharge diagnosis of liver cirrhosis or other liver diseases before the date of ICH. We computed odds ratios for ICH by conditional logistic regressions, adjusting for a number of confounding factors.

**Results:**

There was an increased risk of ICH for patients with alcoholic liver cirrhosis (adjusted OR = 4.8, 95% CI: 2.7–8.3), non-alcoholic liver cirrhosis (adjusted OR = 7.7, 95% CI: 2.0–28.9) and non-cirrhotic alcoholic liver disease (adjusted OR = 5.4, 95%CI:3.1–9.5) but not for patients with non-cirrhotic non-alcoholic liver diseases (adjusted OR = 0.9, 95%CI:0.5–1.6). The highest risk was found among women with liver cirrhosis (OR = 8.9, 95%CI:2.9–26.7) and for patients younger than 70 years (OR = 6.1, 95%CI:3.4–10.9). There were no sex- or age-related differences in the association between other liver diseases (alcoholic or non-alcoholic) and hospitalisation with ICH.

**Conclusion:**

Patients with liver cirrhosis and non-cirrhotic alcoholic liver disease have a clearly increased risk for ICH.

## Background

Spontaneous intracerebral haemorrhage (ICH) is bleeding into the parenchyma of the brain that may extend into the ventricular system. ICH accounts for 10–15% of all cases of stroke and less than 50% are alive after 12 months [1] with many surviving patients suffering from hemiparesis and aphasia [2].

The most important risk factor for ICH is hypertension [3], but age, male gender, diabetes, smoking, antithrombotic treatment, and drug abuse may also increase the risk [4,5]. It is a frequent notion that liver patients are at higher risk for ICH. However, few studies have examined the role of liver cirrhosis and other liver diseases as possible risk factors. Most of the reports are case series without controls [6-9], and the results are conflicting.

Liver diseases may be risk factors for ICH due to impaired coagulation. High alcohol intake is an important risk factor for development of liver diseases and alcohol intake may further induce hypertension. In addition, alcohol intake is associated with changes in the coagulation system and may also affect the integrity of cerebral vessels [4,10-14].

Thus, it remains unresolved whether cirrhosis and other liver diseases are risk factors for ICH. Therefore, we conducted a population-based case-control study in order to examine the association of liver cirrhosis and other liver diseases with the risk of hospitalisation for ICH.

## Methods

This study was conducted within North Jutland, Viborg and Aarhus counties, Denmark with a population of 1.4 million from January 1, 1991, through 31 December 31, 2003. The National Health Service provides tax-supported health care for all inhabitants, allowing free access to general practitioners and hospitals, and it refunds a variable proportion of the costs of prescribed drugs. Using the civil registry number, unique to every Danish citizen and encoding gender and date of birth, an individual and complete hospital discharge and prescription history can be established, and unambiguously linked to population-based registers.

The ethics system in Denmark only deals with biological testing of substances or devices in patients. An approval was therefore not required for our study according to Danish law as it was a register based study. The study was, however, approved by the National Data Protection Agency."

### Cases

The hospital discharge registries retain data on all discharges from all somatic hospitals since 1977 [15]. The files include the civil registry number, date of discharge, and up to 20 discharge diagnoses and procedures, coded according to the International Classification of Diseases, 8th revision until the end of 1993, and 10th revision thereafter [15].

From the discharge history we identified patients with a first-time discharge diagnosis of ICH (ICD8 431.00, 431.08–431.90, 431.98, 431.99; ICD10 I61.0–9) (n = 4,105). Data on drug use was available from 1991 for North Jutland, from 1998 for Viborg and from 1996 for Aarhus. We excluded patients who had not been residents within the counties for at least one year or were younger than 18 years or older than 90 years of age (n = 583). In total, 3,522 cases were available for analyses, of which 2,987 (85%) were discharged from hospitals with in-house computed tomography (CT) or magnetic resonance imaging (MRI).

### Population controls

From the Civil Registration System we selected 10 random controls for each case, matching on age (same date of birth), sex and county. We used the risk-set sampling [16], i.e. the controls had to be alive (at risk for first ICH) at the time the corresponding case was diagnosed. Overall, we included 35,173 controls.

### Liver diseases and possible confounders

Data were obtained from the hospital discharge registries and included diagnoses of liver cirrhosis (ICD-8 code 571.09, 571.90, 571.91, 571.92, 571.99; ICD-10 code K70.3, K74.3, K74.4, K74.5), other non-cirrhotic alcoholic liver disease (ICD-8 code 571.10, 571.11; ICD-10 code K70.0, K70.1, K70.4, K70.9) and non-cirrhotic non-alcoholic liver disease (ICD-8 code 570.00–571.08, 571.12–571.89, 571.93–571.98, 572.00–573.09, ICD-10 code K70.2, K70.5–K70.8, K71.0–K74.2, K47.7–K77.8).

Data on selected confounding factors for cases and controls were obtained from the discharge and prescription databases. These were hospital discharge diagnoses of hypertension, chronic bronchitis and emphysema (used as a proxy for smoking), and diabetes mellitus before or during the admission for ICH. Data on drug usage were obtained from the prescription databases. We collected data on the following medication groups filled within 90 d before the ICH among cases or on the corresponding date among controls: antihypertensive agents (ACE-inhibitors, beta-blockers, calcium antagonists, diuretics and angiotensin II receptor antagonists); insulin or oral hypoglycemic agents; platelet inhibitors (low-dose aspirin, dipyridamol and clopidogrel); high-dose aspirin (200–500 mg); statins; other lipid-lowering drugs; oral anticoagulants (warfarin and phenprocoumon); and, for women, hormone replacement therapy. All of the drugs, except aspirin, were available only by prescription. Pensioners and regular users of aspirin are registered in the databases.

### Statistical analysis

We computed odds ratios (OR) for ICH among hospitalised patients with liver cirrhosis and other non-cirrhotic liver diseases by conditional logistic regressions. First, we computed crude ORs and then ORs adjusted for the confounders, included as dichotomous variables. Analyses were stratified according to sex, age and in-house access to CT of MRI. Estimation of 95% confidence intervals (95% CI) for ORs was based on large sample theory for conditional maximum likelihood estimators.

## Results

Characteristics of the 3522 cases with ICH and 35,173 controls are presented in Table 1. A history of previous hospital admissions for hypertension (p < 0.0001) and diabetes (p < 0.0001) was more frequent among cases than among controls (Table 1). Further, a higher proportion of cases than controls had used the examined drugs (p < 0.05) except for statins and hormone replacement therapy.

**Table 1:** Descriptive characteristics of 3522 cases with ICH and 35,173 controls (matched on age and sex) from North Jutland, Viborg and Aarhus counties, Denmark, 1991–2003

	Cases	Controls
No.	3522	35,173
Age, years*	67 (18–89)	67 (18–89)
Sex		
-Men (%)	1852 (52.6)	18,492 (52.6)
-Women (%)	1670 (47.4)	16,681 (47.4)
*Discharge diagnosis:*		
Cirrhosis (%)†	23 (0.7)	44 (0.1)
Other non-cirrhotic alcoholic liver diseases (%)†	23 (0.7)	41 (0.1)
Other non-cirrhotic non-alcoholic liver diseases (%)†	14 (0.4)	119 (0.3)
Hypertension (%)†	586 (16.6)	2563 (7.3)
Diabetes mellitus (%)†	220 (6.3)	1252 (3.6)
Chronic bronchitis and emphysema (%)†	130 (3.7)	1119 (3.2)
*Prescription for:*		
Antihypertensive drugs (%)#	1603 (45.5)	13,107 (37.3)
Insulin and oral hypoglycemic drugs (%)#	245 (7.0)	1715 (4.9)
Platelet inhibitors (%)#	414 (11.8)	2463 (7.0)
High-dose aspirin (%)#	319 (9.1)	2117 (6.0)
Oral anticoagulants (%)#	173 (4.9)	558 (1.6)
Statins (%)#	134 (3.8)	1324 (3.8)
Other lipid-lowering drugs (%)#	23 (0.7)	148 (0.4)
Hormone replacement therapy (%)#	396 (11.2)	4107 (11.7)

*Mean (range)

†A discharge diagnosis before the admission for ICH.

#Received at least one prescription within 90 days (platelet inhibitors, high-dose aspirin, oral anticoagulants), ever received at least one prescription (insulin and hypoglycemic drugs, statins, other lipid-lowering drugs, hormone replacement therapy) or ever received at least three prescriptions (antihypertensive drugs).

Most patients with cirrhosis had alcoholic cirrhosis (58/67, 87%). Most patients with non-cirrhotic alcoholic liver disease had alcoholic steatosis (30/56, 53%) or unspecified liver diseases associated with a history of alcohol abuse (18/56, 32%). Among the patients with non-cirrhotic non-alcoholic liver disease, the majority had hepatitis, acute, subacute or chronic, (60/133, 45%) or other unspecified liver diseases (58/133, 44%).

There was a five-fold increased risk for ICH among patients with a diagnosis of liver cirrhosis or non-cirrhotic alcoholic liver disease (Table 2). Both alcoholic and non-alcoholic cirrhosis were associated with an increased risk of ICH (Table 2).

**Table 2:** Crude and adjusted ORs for ICH according to a diagnosis of liver disease before the date of hospitalisation.

	Cases (n = 3522)	Controls (n = 35,173)	Crude OR (95% CI)	Adjusted OR* (95% CI)
Liver cirrhosis				
No	3499	35,129	1.0	1.0
Yes	23	44	5.2 (3.2–8.7)	5.1 (3.1–8.5)
Alcoholic cirrhosis				
No	3503	35,134	1.0	1.0
Yes	19	39	4.9 (2.8–8.5)	4.8 (2.7–8.3)
Non-alcoholic cirrhosis				
No	3518	35,168	1.0	1.0
Yes	4	5	8.0 (2.2–29.9)	7.7 (2.0–28.9)
Non-cirrhotic alcoholic liver diseases				
No	3501	35,138	1.0	1.0
Yes	21	35	6.0 (3.5–10.3)	5.4 (3.1–9.5)
Non-cirrhotic non-alcoholic liver diseases				
No	3508	35,054	1.0	1.0
Yes	14	119	1.2 (0.7–2.0)	0.9 (0.5–1.6)

* Adjusted for discharge diagnoses of hypertension, diabetes mellitus, chronic bronchitis and emphysema and prescriptions for antihypertensive agents, insulin or oral hypoglycaemic agents, platelet inhibitors, high-dose aspirin, oral anticoagulants, statins, other lipid-lowering agents and hormone replacement therapy.

In contrast, a diagnosis of non-alcoholic non-cirrhotic liver disease was not associated with increased risk.

The shortest time interval between the diagnosis of liver disease and hospitalisation with ICH was found among patients with alcoholic cirrhosis (median time = 2.3 years, range: 0.05–22.0 years) and non-cirrhotic alcoholic liver disease (median time = 4.5 years, range: 0.2–25.1 years). The corresponding time intervals were 6.2 years (range:0.06–14.2 years) and 6.3 years (range:0.03–24.9 years) for patients with non-alcoholic liver cirrhosis and patients with non-cirrhotic non-alcoholic liver disease, respectively.

The risk of ICH in cirrhosis patients was highest among women and patients younger than 70 years (Table 3). There were no sex- or age-related differences in the association between other liver diseases (alcoholic or non-alcoholic) and ICH (data not shown).

**Table 3:** Crude and adjusted ORs for ICH according to a diagnosis of liver cirrhosis.

	Cases*	Controls*	Crude OR (95% CI)	Adjusted OR (95% CI) *
Sex				
Women	6/1670	7/16,681	8.6 (2.9–25.5)	8.9 (2.9–26.7)
Men	17/1852	37/18,492	4.6 (2.6–8.2)	4.3 (2.4–7.7)
Age				
<70 years	20/1710	31/17,173	6.6 (3.8–11.7)	6.1 (3.4–10.9)
≥ 70 years	3/1812	13/18,000	2.1 (0.6–7.6)	2.2 (0.6–8.0)

* Proportion with liver cirrhosis.

† Adjusted for discharge diagnoses of hypertension, diabetes mellitus, chronic bronchitis and emphysema and prescriptions for antihypertensive agents, insulin or oral hypoglycaemic agents, platelet inhibitors, high-dose aspirin, oral anticoagulants, statins, other lipid-lowering agents and hormone replacement therapy.

Restricting the analysis to hospitals with access to CT or MR did not change the overall findings. Adjusted ORs were 5.6 (95% CI: 3.3–9.5), 5.9 (95% CI: 3.2–10.8) and 1.1 (95% CI: 0.6–1.9) for patients with liver cirrhosis, other non-cirrhotic alcoholic liver disease and other non-cirrhotic non-alcoholic liver disease, respectively. The number of cases with liver disease hospitalised on hospitals without easy access to brain imaging facilities was too small to allow a separate multivariate analysis.

For comparison, we also computed the adjusted relative risk estimates for other well-established risk factors for ICH including a discharge diagnosis of hypertension (adjusted OR = 2.2, 95% CI: 2.0–2.4) and use of oral anticoagulants (adjusted OR = 2.9, 95% CI: 2.5–3.5) and platelet inhibitors (adjusted OR = 1.7, 95% CI: 1.5–1.9).

## Discussion

In this population-based case-control study we found a markedly increased risk for ICH among hospitalised patients with liver cirrhosis or non-cirrhotic alcoholic liver disease, while non-cirrhotic non-alcoholic liver disease did not increase the risk of ICH.

The strengths of the study are its size, its population-based design and its ability to control for other risk factors to ICH. The recording of exposure and confounder data into the administrative registries before admission for ICH excludes any risk of recall bias. The main limitation is the use of routine hospital discharge diagnoses. It is well-known that misclassification do occur in hospital discharge registries and we cannot exclude the possibility that miscoding may also have occurred in this study, in particular for conditions mimicking spontaneous ICH, including subarachnoid hemorrhage, hemorrhagic brain infarction and traumatic brain injuries. However, the overall high validity of the ICH diagnosis appear to be high [17-19] although variation between specialized and non-specialized departments have been reported [18,20]. In a random sample study we have shown a high predictive value of 90% (95% CI: 81–95%) of the diagnosis in departments with in-house CT or MRI scanners [21]. Restricting the analyses to departments with easy access to brain imaging facilities did not substantially change the results in our study, which may indicate that miscoding was not a major problem in our study. In focusing on hospitalised patients we may have missed patients with discrete ICH or patients who died before reaching hospital. Old people and patients with a short life expectancy may have been less likely to be hospitalised. However, the age distribution in our study (mean 67 years, range 18–89 years) shows that also very senior citizens with ICH were hospitalised.

Finally, it should be noted that the sensitivity of some of the hospital discharge diagnoses used in our study was modest (e.g., only 16.6% of the cases and 7.3% of the controls were registered with a hypertension diagnosis). To compensate for this well-known limitation of hospital discharge registries, we obtained data on the prescription history of both cases and controls. The prescription data revealed that a much higher proportion of both cases and controls in fact suffered from hyptertension (i.e., 45% and 38% of cases and controls, respectively, used antihypertensives). The overall validity of our data was supported by the fact that we found adjusted relative risk estimates for well established ICH risk factors, including a discharge diagnosis of hypertension and use of oral anticoagulants and platelet inhibitors that were similar to those previously reported in the literature [3,22].

The increased risk of ICH for patients with liver cirrhosis or alcoholic non-cirrhotic liver diseases is in accordance with several case series [6-8,23]. Other studies indicate that liver disease may also be associated with haematoma enlargement, a major cause of clinical deterioration [24,25]. In contrast, autopsy studies do not shown any relation between liver cirrhosis and ICH, but rather report a reduced risk of all thromboembolic events in cirrhosis patients [26,27]. Likewise, there was no case of spontaneous ICH in a case series of 100 patients with coagulopathic liver disease admitted for non-traumatic acute mental changes [28].

A recent prospective study demonstrated discretely elevated serum aminotransferases to be a predictor for ICH (OR = 2.89 (2.09–4.01) [29] even after controlling for alcohol intake, and also so in non-drinkers. This suggests that even slight liver impairment may be a risk factor for ICH independent of confounding by alcohol. A recent in-vitro study on tissue from cirrhosis patients showed damaged vessel walls and deficient platelet aggregation [30]. Such mechanism could be involved in the pathogenesis of ICH and contribute towards the association of liver cirrhosis with ICH along with other changes in the coagulation system in cirrhosis patients. A recent case-control study from Chile found a higher proportion of ICH patients with liver diseases compared to controls, however, liver diseases were not well defined [31] and no significant association between liver disease and ICH was demonstrated from the stroke registry of Dijon [32].

In the context of this study, the effects of alcohol are particularly important. Alcohol consumers have increased risk of stroke as documented in several case-control, cross-sectional and cohort studies. High alcohol intake increases the risk of all stroke subtypes, ischemic and hemorrhagic, while light to moderate alcohol intake protects, primarily against ischemic stroke of atherothrombotic origin [13,33-35]. The relation between high alcohol intake and ICH may involve several mechanisms among which alcohol-induced hypertension and coagulation disorders – with or without cirrhosis – are the most likely. The relation seems to be more complex when involving cirrhosis. A previous clinical study did not find an increased risk of alcohol-induced hypertension or ICH in cirrhosis patients [36]. In contrast binge vs. regular drinking seems to induce more severe hypertension [37]. In our study, liver cirrhosis was almost entirely alcohol-related, which may contribute to the association between liver cirrhosis and ICH in that group. However, both cirrhosis of alcoholic and non-alcoholic aetiology was associated with the same increased risk, suggesting the high risk to be related to cirrhosis as such, in accordance with the conclusion of a review by Olson [38]. The non-cirrhotic alcoholic liver diseases were associated with the same risk as was cirrhosis, whereas there was not increased risk for non-cirrhotic non-alcoholic liver disease.

These findings give some support to the hypothesis of alcohol being part of the mechanism of the association between liver diseases and ICH although they are by no way definitive. There may be additive or synergistic risks due to the effects of alcohol and of cirrhotic coagulopathy. It should be noted that the present data set using hospital discharge diagnoses cannot be used to elucidate the effects of recent alcohol intake on the risk of ICH.

## Conclusion

Patients with alcoholic and non-alcoholic liver cirrhosis and non-cirrhotic alcoholic liver disease had a substantially increased risk for ICH. The findings, while not excluding a risk mediated by alcohol, suggest an independent risk due to liver cirrhosis.

## Competing interests

The authors declare that they have no competing interests.

## Authors' contributions

HG, SPJ, PJ, HV, UT-J, HTS: Conception and design, acquisition of data and interpretation of data as well as being involved in drafting the manuscript and/or revising it critically for important intellectual content. MG: Did the statistical analysis. All authors have read and approved the final manuscript.

## Pre-publication history

The pre-publication history for this paper can be accessed here:


